# The emergence and circulation of human immunodeficiency virus (HIV)-1 subtype C

**DOI:** 10.1099/jmm.0.001827

**Published:** 2024-05-17

**Authors:** Xingguang Li, Sana Tamim, Nídia S. Trovão

**Affiliations:** 1 Guoke Ningbo Life Science and Health Industry Research Institute, Ningbo, 315000, PR China; 2 Division of International Epidemiology and Population Studies, Fogarty International Center, National Institutes of Health, Bethesda, Maryland, 20892, USA

**Keywords:** HIV-1, maximum likelihood, phylodynamics, subtype C, viral introductions

## Abstract

**Introduction.** Human immunodeficiency virus (HIV)-1 subtype C is the most prevalent globally and is thought to have originated in non-human primates in the Democratic Republic of Congo.

**Hypothesis/Gap Statement.** Although the global dominance of HIV-1 subtype C is well established, a thorough understanding of its evolutionary history and transmission dynamics across various risk populations remains elusive. The current knowledge is insufficient to fully capture the global diversification and dissemination of this subtype.

**Aim.** We for the first time sought to investigate the global evolutionary history and spatiotemporal dynamics of HIV-1 subtype C using a selection of maximum-likelihood-based phylodynamic approaches on a total of 1210 near full-length genomic sequences sampled from 32 countries, collected in 4 continents, with sampling dates between 1986–2019 among various risk groups were analysed.

**Methodology.** We subsampled the HIV-1 subtype C genomic datasets based on continent and risk group traits, and performed nucleotide substitution model selection analysis, maximum likelihood (ML) phylogenetic reconstruction, phylogenetic tree topology similarity analysis, temporal signal analysis and traced the timings of viral spread both geographically and by risk group.

**Results.** Based on the phylodynamic analyses of four datasets (full1210, locrisk626, loc562 and risk393), we inferred the time to the most recent common ancestor (TMRCA) in the 1930s and an evolutionary rate of 0.0023 substitutions per site per year. The total number of introduction events of HIV-1 subtype C between continents and between risk groups is estimated to be 71 and 115, respectively. The largest number of introductions occurred from Africa to Europe (*n*=32), from not-recorded to heterosexual (*n*=40) and from heterosexual to not-recorded (*n*=51) risk groups.

**Conclusion.** Our results emphasize that HIV subtype C has mainly spread from Africa to Europe, likely through heterosexual transmission.

## Introduction

Patients infected with HIV (human immunodeficiency virus) can progress to have an infectious disease designated as AIDS, which is one of the world’s most fatal infectious diseases, particularly across sub-Saharan Africa [1], resulting in heightened risk of life-threatening conditions that constitute a global public health burden. There were 36.8 million people infected with HIV in 2019 around the world [2]. The number of people infected with HIV in the African region, region of the Americas, South-East Asia region, European region, Western Pacific region and Eastern Mediterranean region were 25.99, 3.95, 2.92, 2.22, 1.42 and 0.32 million, respectively. There were 1.99 million new infections of HIV/AIDS in 2019, and 863 837 deaths from HIV/AIDS worldwide [3]. In some countries in sub-Saharan Africa, such as South Africa, Botswana and Mozambique, HIV/AIDS is the leading cause of death [4]. Unprotected sex is among the top six leading risk factors for death in sub-Saharan Africa [1]. The number of people who receive anti-retroviral treatment (ART) has increased significantly, from 2 million people in 2005 to 23 million in 2018 [1], and from 73 % of people living with HIV received ART in 2020 to 76 % in 2022 [5]. Furthermore, there are estimates that the price tag for providing long-term HIV/AIDS prevention and treatment in 2015–2050 in the nine sub-Saharan countries most affected by the epidemic ranges from $98 billion at current coverage levels to $261 billion if coverage is scaled up [6].

HIV-1 M group is sub-categorized into ten distinct subtypes (A, B, C, D, F, G, H, I, J and K) and numerous circulating recombinant forms (CRFs) [7,8]. HIV-1 subtype C is currently the most prevalent subtype globally and has dominated in India, Southern Africa and Ethiopia, where it is responsible for at least 89 % of infections between 1990 and 2015 [4]. However, its global distribution has changed over time, with increasing its proportion between 1990 and 2009, followed by a decrease between 2010 and 2015 [4]. HIV-1 subtype C is estimated to represent 48–50 % of infections between 2000 and 2007 worldwide [9], and 46.6 % of infections between 2010 and 2015 worldwide [4], respectively.

Previous phylogenetic analysis of 346 partial *pol* sequences suggests that HIV-1 subtype C originated from a non-human primate (NHP) in Mbuji-Mayi, Southern Democratic Republic of Congo in the 1950s [10]. The ancestral lineages were transmitted to Zimbabwe, Ethiopia, Kenya, Tanzania and Uganda likely through migration of mineworkers returning from the Democratic Republic of Congo (DRC) [10,12]. Complex socio-political change in South Africa led to multiple introductions of subtype C from neighbouring countries Botswana, Malawi, Mozambique, Tanzania, Zambia and Zimbabwe and disseminated to eastern and southern Africa [13]. Most HIV-1 subtype C cases are caused by sexual transmission [14].

In order to gain a more comprehensive understanding of the evolutionary and transmission dynamics of HIV-1 subtype C, we performed comprehensive and detailed phylodynamic analyses of 1221 near full-length genomic sequences (HXB2 Genome Position 790–9417, with a minimum fragment length of 6 000 bp) of HIV-1 subtype C with a known sampling of time, geographic location and risk group. Our findings suggest that subsampling simultaneously by location, risk group and date produces the most accurate reconstruction of the dynamics of HIV-1 subtype C. This work suggests that HIV-1 subtype C likely emerged earlier than previously estimated and that its transmission dynamics were punctuated by viral spread from Africa to Europe through heterosexual transmission.

## Methods

### Sequence dataset compilation

All available HIV-1 subtype C near-complete genome sequences (HXB2 genome position 790–9417, with minimal fragment length of 6000 nt) with known sampling dates and geographic information were retrieved from the Los Alamos National Laboratory (LANL) HIV Sequence Database [15] as of 26 March 2021. Problematic sequences (for instance, high content of non-ACTG characters; likely contamination with a laboratory strain; a sequence containing an artifactual deletion of >100 nucleotides; small sequences with length <50 bp; and sequences deposited as the reverse complement DNA strand), as defined by LANL [16] were removed, and only one sequence per patient was selected before download. Sequence quality was analysed using the Quality Control tool [17], the genotype assignment of all sequences was confirmed using RIP v3.0 [18] and hypermutation analysis was performed using Hypermut v2.0 [19] from the LANL website. The final dataset included 1221 publicly available near-complete genome sequences of HIV-1 subtype C (full1221) with known sampling dates between 1986 and 2019 across 32 countries (Table S1). We used the two-letter codes for each country [20]. We grouped the risk group data associated with the sequences in full1221 into six categories, male sex with male (SM), IV drug user (PI), heterosexual (SH), mother-baby (MB), not recorded (NR) and the other (OT) [21].

### Subsampling strategy

Multiple sequence alignments of the full1221 dataset were performed using MAFFT v7.427 [22] under default parameters and then adjusted manually in BioEdit v7.2.5 [23]. Subsequently, we excluded sequences with fewer than 50 % nucleotides and duplicate sequences, defined as having the same collection date, country, risk group and nucleotide sequence. The result is a full genome dataset of 1210 sequences (full1210).

Further subsampling was performed with Sequence sampling tool for phylogenetics (SAMPI) [24] to obtain a homogenous collection of samples per country, risk group and date (date, country and risk group; date and country; and date and risk group), as described elsewhere [25]. This resulted in full genome datasets of 626 sequences subsampled based on sampling location, risk group and collection date (locrisk626); 562 sequences subsampled based on sampling location and collection date (loc562); and 393 sequences subsampled based on risk group and collection date (risk393). This resulted in full genome datasets of 626 (locrisk626), 562 (loc562) and 393 (risk393) sequences ranging from 329 to 503 sequences shared among them (Table S2).

### Nucleotide substitution model selection and phylogenetic construction

The best-fit nucleotide substitution model for the four datasets (full1210, locrisk626, loc562 and risk393) was performed according to the Akaike Information Criterion (AIC), Corrected Akaike Information Criterion (AICc), Bayesian Information Criterion (BIC) and Decision Theory Performance-based Selection (DT) with 3 (24 candidate models) and 11 (88 candidate models) substitution schemes in jModelTest v2.1.10 [26]. The general time-reversible substitution model (GTR) with among-site variation (+Γ4) and a proportion of invariable sites (+I), designated as GTR+Γ4+I, was chosen as the best-fit nucleotide substitution model for the four datasets (full1210, locrisk626, loc562 and risk393) according to the four calculations (AIC, AICc, BIC and DT) and the two substitution schemes (three and eight). Multiple iterations of maximum-likelihood (ML) phylogeny reconstruction using RAxML v8.2.12 [27] under a GTR+Γ_4_+I nucleotide substitution model with 1000 bootstrap replicates [28] were performed for the four datasets (full1210, locrisk626, loc562 and risk393).

### Evolutionary rate and time origin estimates

To estimate the evolutionary rate and origin time for the ML phylogenies of the four datasets (full1210, locrisk626, loc562 and risk393), we employed a linear regression of root-to-tip genetic distances against sampling dates in TempEst v1.5.3 [29], ML dating in TreeTime [30], least-squares dating in LSD2 [31] and a Gamma-Poisson mixture model in treedater [32].

### Viral introduction analysis

A migration model implemented in TreeTime [30] was fitted on the resulting time-scaled tree topology generated using ML dating in TreeTime [30] for the four datasets (full1210, locrisk626, loc562 and risk393). Its ‘migration’ function allowed reconstructing the geographic locations and risk groups from tips to internal nodes. The resulting annotated time-scaled tree topology was used to infer the number of viral introductions into different geographic locations and risk groups through time.

## Results

### Global distribution of HIV-1 subtype C genome sequences

**Fig. 1.**
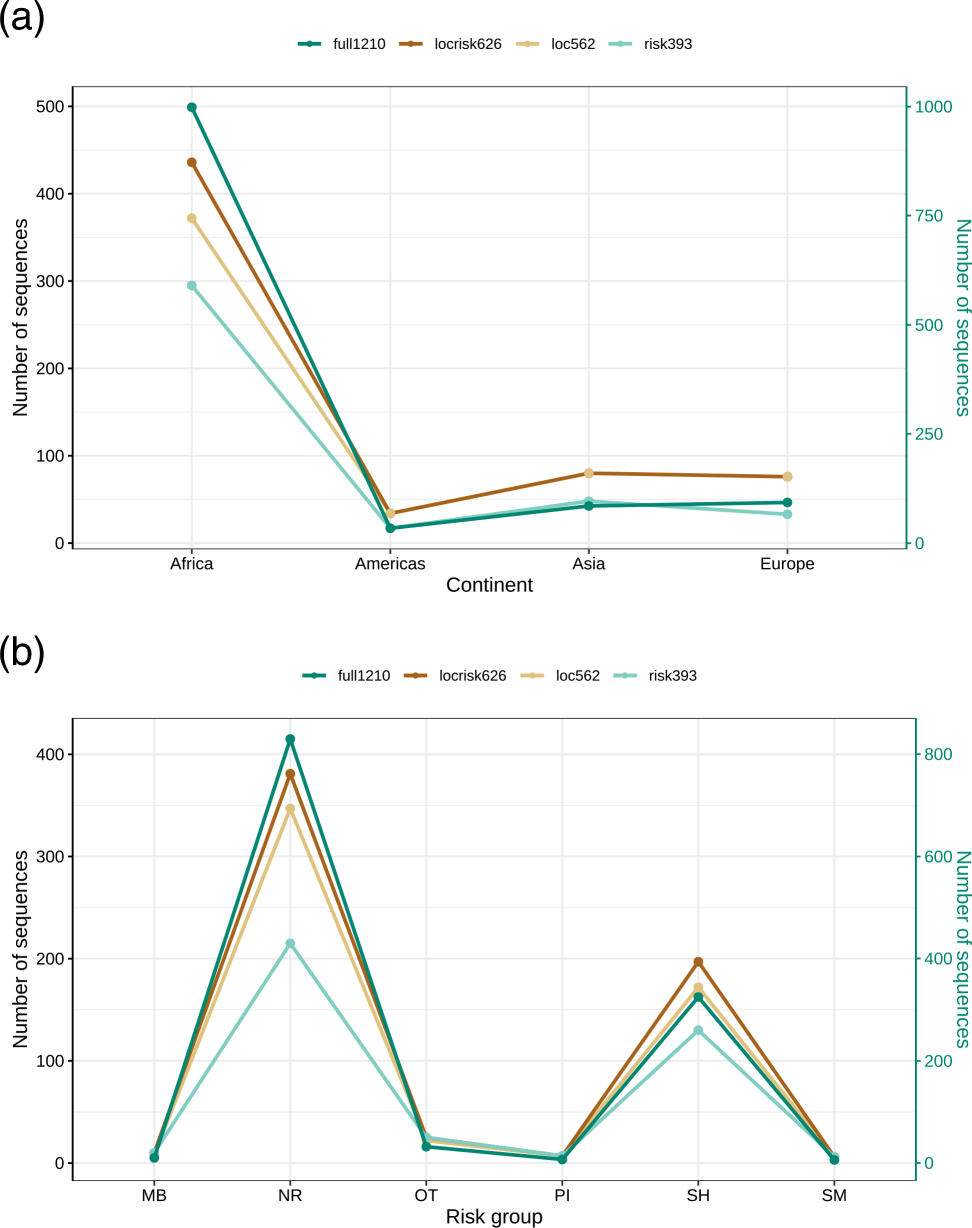
Continent and risk group distributions of HIV-1 subtype C. (**a**) Continent distribution for the four datasets (full1210, locrisk626, loc562 and risk393). (**b**) Risk group distribution for the four datasets (full1210, locrisk626, loc562 and risk393). SM: male sex with male; PI: IV drug user; SH: heterosexual; MB: mother-baby; NR: not recorded; and OT: the other.

The full1210 dataset included 1210 full genome sequences of HIV-1 subtype C from 32 countries (Fig. S1, available in the online version of this article): Argentina (AR; *n*=1), Belgium (BE; *n*=2), Bulgaria (BG; *n*=1), Brazil (BR; *n*=23), Botswana (BW; *n*=52), China (CN; *n*=8), Cyprus (CY; *n*=11), Germany (DE; *n*=1), Denmark (DK; *n*=1), Spain (ES; *n*=7), Ethiopia (ET; *n*=22), United Kingdom (GB; *n*=37), Georgia (GE; *n*=1), Israel (IL; *n*=5), India (IN; *n*=44), Kenya (KE; *n*=5), Myanmar (MM; *n*=1), Mongolia (MW; *n*=28), Nigeria (NG; *n*=2), Nepal (NP; *n*=12), Pakistan (PK; *n*=1), Sweden (SE; *n*=44), Senegal (SN; *n*=2), Somalia (SO; *n*=1), Thailand (TH; *n*=1), Tanzania (TZ; *n*=59), Uganda (UG; *n*=7), United States (US; *n*=9), Uruguay (UY; *n*=1), Yemen (YE; *n*=1), South Africa (ZA; *n*=601), and Zambia (ZM; *n*=219) with known sampling date between 1986 and 2019. As shown in Fig. 1, the samples are classed into four continents: Africa (*n*=998), Americas (*n*=34), Asia (*n*=85) and Europe (*n*=93), and six risk groups: MB (*n*=10), NR (*n*=830), OT (*n*=32), PI (*n*=7), SH (*n*=325) and SM (*n*=6). The samples are primarily from South Africa (ZA; 601/1210, 49.7 %) and Zambia (ZM; 219/1210, 18.1 %), and among NR (830/1210, 68.6 %) and SH (325/1210, 26.9 %).

The locrisk626 dataset included 626 full genome sequences of HIV-1 subtype C from 32 countries: AR (*n*=1), BE (*n*=2), BG (*n*=1), BR (*n*=23), BW (*n*=36), CN (*n*=8), CY (*n*=11), DE (*n*=1), DK (*n*=1), ES (*n*=7), ET (*n*=12), GB (*n*=20), GE (*n*=1), IL (*n*=5), IN (*n*=39), KE (*n*=5), MM (*n*=1), MW (*n*=28), NG (*n*=2), NP (*n*=12), PK (*n*=1), SE (*n*=44), SN (*n*=2), SO (*n*=1), TH (*n*=1), TZ (*n*=58), UG (*n*=7), US (*n*=9), UY (*n*=1), YE (*n*=1), ZA (*n*=195) and ZM (*n*=90) with known sampling date between 1986 and 2019. As shown in Fig. 1, the samples are classed into four continents: Africa (*n*=436), Americas (*n*=34), Asia (*n*=80) and Europe (*n*=76), and six risk groups: MB (*n*=10), NR (*n*=384), OT (*n*=25), PI (*n*=7), SH (*n*=197), and SM (*n*=6). The samples were primarily from ZA (195/626, 31.2 %) and ZM (90/626, 14.4 %), and among NR (381/626, 60.9 %) and SH (197/626, 31.5 %).

The loc562 dataset included 562 full genome sequences of HIV-1 subtype C from 32 countries: AR (*n*=1), BE (*n*=2), BG (*n*=1), BR (*n*=23), BW (*n*=32), CN (*n*=8), CY (*n*=11), DE (*n*=1), DK (*n*=1), ES (*n*=7), ET (*n*=12), GB (*n*=20), GE (*n*=1), IL (*n*=5), IN (*n*=39), KE (*n*=5), MM (*n*=1), MW (*n*=26), NG (*n*=2), NP (*n*=12), PK (*n*=1), SE (*n*=44), SN (*n*=2), SO (*n*=1), TH (*n*=1), TZ (*n*=58), UG (*n*=7), US (*n*=9), UY (*n*=1), YE (*n*=1), ZA (*n*=151) and ZM (*n*=76) with known sampling date between 1986 and 2019. As shown in Fig. 1, the samples are classed into four continents: Africa (*n*=372), Americas (*n*=34), Asia (*n*=80) and Europe (*n*=76), and six risk groups: MB (*n*=8), NR (*n*=347), OT (*n*=22), PI (*n*=7), SH (*n*=172) and SM (*n*=6). The samples were primarily from ZA (151/562, 26.9 %) and ZM (76/562, 13.5 %), and among NR (347/562, 61.7 %) and SH (172/562, 30.6 %).

The risk393 dataset included 393 full genome sequences of HIV-1 subtype C from 29 countries: BE (*n*=1), BG (*n*=1), BR (*n*=7), BW (*n*=33), CN (*n*=6), CY (*n*=5), DE (*n*=1), DK (*n*=1), ES (*n*=5), ET (*n*=1), GB (*n*=14), IL (*n*=5), IN (*n*=25), KE (*n*=2), MM (*n*=1), MW (*n*=7), NG (*n*=1), NP (*n*=4), PK (*n*=1), SE (*n*=10), SN (*n*=2), SO (*n*=1), TZ (*n*=49), UG (*n*=7), US (*n*=9), UY (*n*=1), YE (*n*=1), ZA (*n*=137) and ZM (*n*=55) during 1986–2009. As shown in Fig. 1, the samples are classed into four continents: Africa (*n*=295), Americas (*n*=17), Asia (*n*=48) and Europe (*n*=33), and six risk groups: MB (*n*=10), NR (*n*=215), OT (*n*=25), PI (*n*=7), SH (*n*=130) and SM (*n*=6). The samples were primarily from ZA (137/393, 34.9 %) and ZM (55/393, 14.0 %), and among NR (215/393, 54.7 %) and SH (130/393, 33.1 %).

### Evolutionary dynamics of HIV-1 subtype C using full and subsampled datasets

**Fig. 2.**
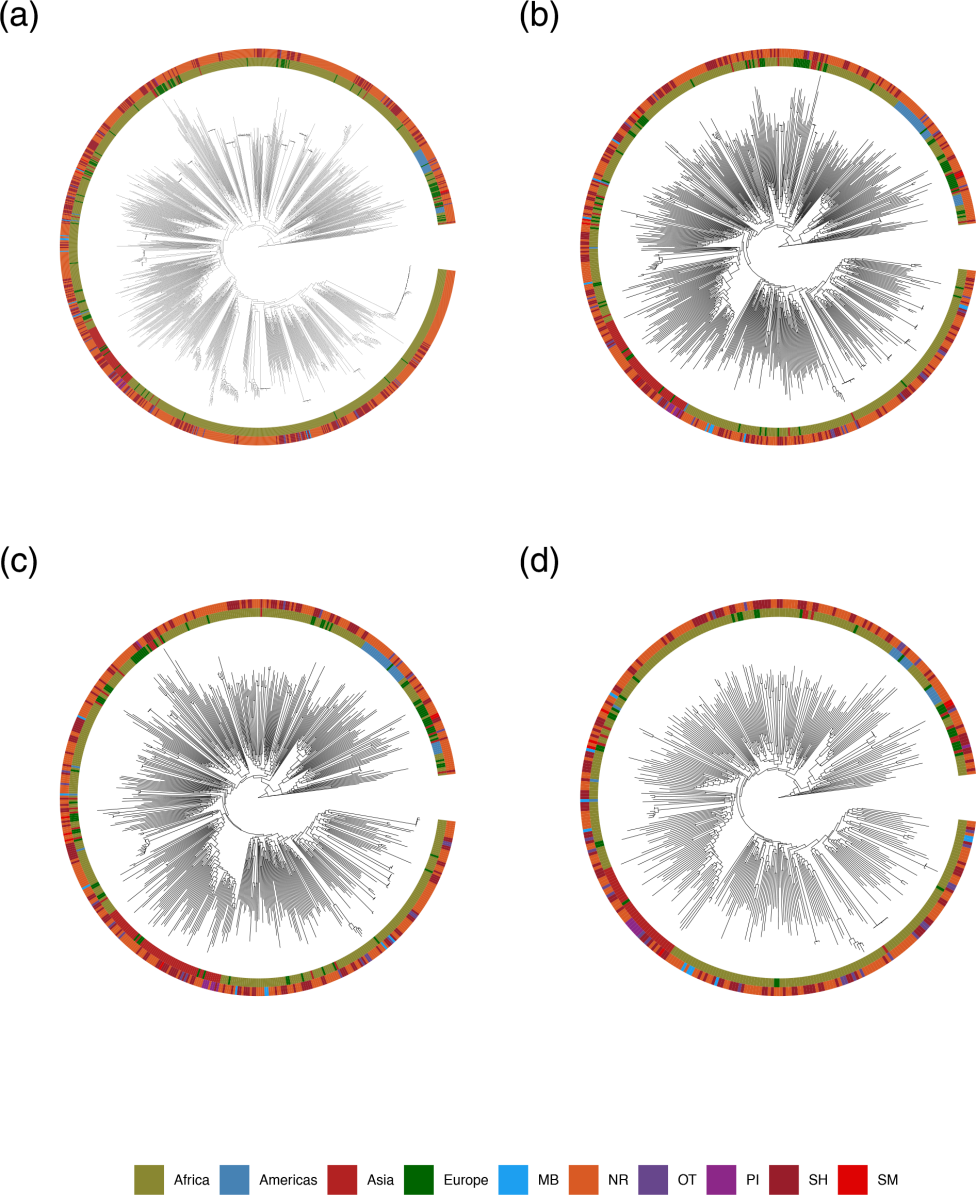
Estimated ML phylogenetic trees of HIV-1 subtype C. ML phylogenetic trees of HIV-1 subtype C for the four datasets (A, B for full1210; B for locrisk626; C for loc562; and D for risk393). The two circles of coloured cells show sampling locations (inner circle) and risk groups (outer circle). SM: male sex with male; PI: IV drug user; SH: heterosexual; MB: mother-baby; NR: not recorded; and OT: the other.

The phylogenies for the four datasets (full1210, locrisk626, loc562 and risk393), inferred from ML inference, suggest that the overall tree topologies are consistent between full1210 and subsampled datasets (locrisk626, loc562 and risk393) (Figs 2 and S2). Linear regression of root-to-tip genetic distances against sampling dates revealed a relatively strong temporal signal (i.e. clock-like evolution) for the four datasets (full1210: correlation coefficient=0.64, R^2^=0.41, *p*-value=0; locrisk626: correlation coefficient=0.61, R^2^=0.37, *p*-value=0; loc562: correlation coefficient=0.64, R^2^=0.41, *p*-value=0; risk393: correlation coefficient=0.60, R^2^=0.36, *p*-value=0), without any clear outlier sequences (Fig. S3). These results suggest relatively clock-like molecular evolution, with estimated evolutionary rates of 2.9×10^−3^, 2.4×10^−3^, 2.5×10^−3^, 2.1×10^−3^ substitutions per site per year, respectively, and the time to the most recent common ancestor (TMRCA) occurring on 23 June 1952, 19 April 1943, 22 March 1949 and 16 May 1936 for full1210, locrisk626, loc562 and risk393, respectively (Table 1). The estimated evolutionary rate and TMRCA using ML dating in TreeTime [30] were 2.9×10^−3^, 2.3×10^−3^, 2.4×10^−3^, 2.1×10^−3^ substitutions per site per year and 6 February 1940, 8 August 1930, 2 July 1933 and 15 March 1925 for full1210, locrisk626, loc562 and risk393, respectively. The estimated evolutionary rate and TMRCA using least-squares dating in LSD2 [31] were 2.0×10^−3^, 2.1×10^−3^, 2.0×10^−3^, 1.9×10^−3^ substitutions per site per year and 13 September 1927, 11 March 1937, 18 November 1936 and 11 January 1928 for full1210, locrisk626, loc562 and risk393, respectively. The estimated evolutionary rate and TMRCA using Gamma-Poisson mixture model in treedater [32] were 2.4×10^−3^, 2.3×10^−3^, 2.4×10^−3^, 2.3×10^−3^ substitutions per site per year and 10 February 1938, 29 June 1939, 17 March 1943 and 19 April 1940 for full1210, locrisk626, loc562 and risk393, respectively.

**Table 1.** Maximum likelihood phylogenetic estimates of TMRCA and evolutionary rate for near full-length genomic sequences of HIV-1 subtype C

Dataset	no. of genomes	TMRCA				Evolutionary rate (substitutions/site/year)			
		TempEst	TreeTime	LSD2	Treedater	TempEst	TreeTime	LSD2	Treedater
full1210	1210	23 June 1952	6 February 1940	13 September 1927	10 February 1938	0.0029	0.0029	0.0020	0.0024
locrisk626	626	19 Apri l 1943	8 August 1930	11 March 1937	29 June 1939	0.0024	0.0023	0.0021	0.0023
loc562	562	22 March 1949	2 July 1933	18 November 1936	17 March 1943	0.0025	0.0024	0.0020	0.0024
risk393	393	16 May 1936	15 March 1925	11 January 1928	19 April 1940	0.0021	0.0021	0.0019	0.0023

### Ancestral trait reconstruction reveals geographic and risk group transitions in HIV-1 subtype C

**Fig. 3.**
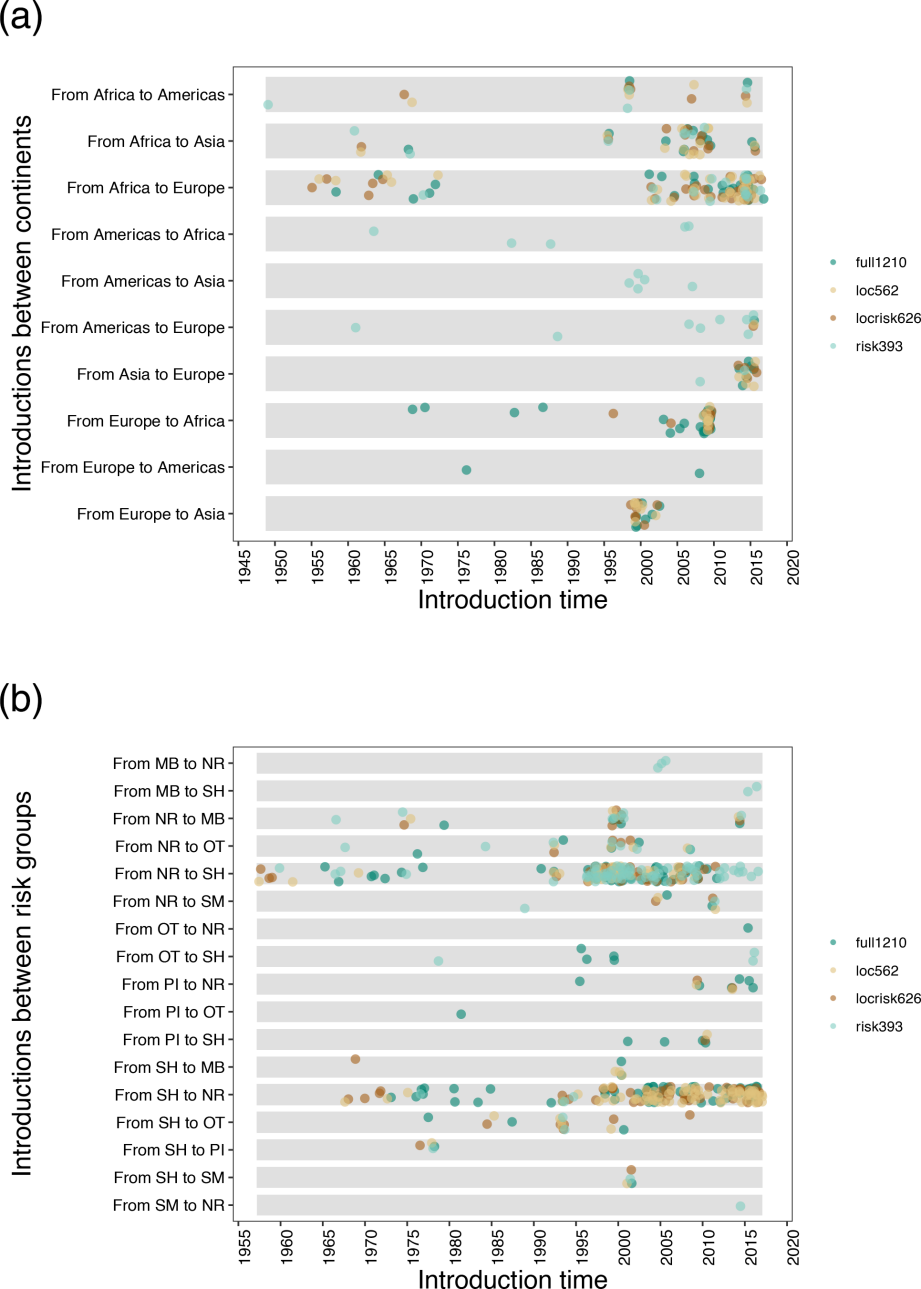
Estimated number of introductions of HIV-1 subtype C through time. (**a**) Estimated number of introductions among continents for the four datasets (full1210, locrisk626, loc562 and risk393) through time. (**b**) Estimated number of introductions among risk groups for the four datasets (full1210, locrisk626, loc562 and risk393) through time. SM: male sex with male; PI: IV drug user; SH: heterosexual; MB: mother-baby; NR: not recorded; and OT: the other.

We performed ancestral trait reconstruction of location and risk group for the four datasets using ML-dated phylogenies obtained in TreeTime [30] and their ‘migration’ function. The total number of viral transitions between continents were 95, 71, 73 and 45 for full1210, locrisk626, loc562 and risk393, respectively. The total number of transmission events between risk groups were 158, 115, 125 and 98 for full1210, locrisk626, loc562 and risk393, respectively. As shown in Fig. S4, when compared to the dynamics inferred for full1210 and risk393, locrisk626 and loc562 have similar patterns both in terms of introduction events among continents and risk groups. Regarding the viral spatial spread, we observed a higher degree of introductions from Europe to Africa for the full1210 dataset when compared to that estimated for locrisk626 and loc562, likely due to sampling bias resulting in an excess of European sequences and/or limited genomic surveillance from Africa. However, no viral introductions from Europe to Africa were estimated for risk393 (Fig. S4A). Notably, risk393 also has the lowest number of introductions from Africa to Europe compared to the estimated for full1210, locrisk626 and loc562 (Fig. S4A), likely due to subsampling irrespective of location. In relation to the introduction events among risk groups, the full1210 and risk393 datasets have higher numbers of introductions from NR to SH compared to the locrisk626 and loc562 datasets (Fig. S4B). However, risk393 has a lower number of introductions from SH to NR compared to full1210, locrisk626 and loc562 (Fig. S4B). As shown in Fig. 3, locrisk626 and loc562 have consistent patterns compared to full1210 and risk393 both for introductions into different continents and risk groups through time.

The spatiotemporal dynamics were characterized by two major waves of viral introduction during 1955–1970 and 1997–2017 from Africa to Europe (Fig. 3a) led by NR to SH (Fig. 3b) risk groups.

## Discussion

Based on a comprehensive ML-based phylodynamic analysis of near full-length genomic sequences of HIV-1 subtype C, we shed light on the viral dynamics leading to outbreaks in different locations among different risk groups around the world while assessing the consistency of these patterns using diverse subsampling schemes.

Our study revealed the presence of relatively strong clock-like evolution for the four datasets. However, estimates of the TMRCA for near full-length genomic sequences of HIV-1 subtype C were not consistent for the four datasets, sometimes with discrepancies of up to more than 16 years between full1210 and risk393. Notably, except when using the linear regression analysis of full1210 in TempEst v1.5.3 [29], which estimated the origin of HIV-1 subtype C to be 23 June 1952, our results using near full-length genomes were not consistent with a previous study [10] that analysed 346 partial *pol* sequences from the DRC, showing that the origin of HIV-1 subtype C was in the mining city of southern DRC, Mbuji-Mayi, in the 1950s. This discrepancy might be due to the limited temporal, risk group and geographical span of the *pol* dataset used in that study. It has also been previously demonstrated that whole genome sequences yield more accurate estimates of the ancestral nodes and the spatial inference [33,34].

The Gamma-Poisson mixture model in treedater [32] had the narrowest interval (from 1938 to 1943) and was within the interval estimated using the linear regression analysis in TempEst v1.5.3 [29] (from 1936 to 1952), which in turn was almost nested within the interval estimated with ML dating in TreeTime [30] (from 1925 to 1940). Estimates of evolutionary rate across all four datasets using linear regression analysis in TempEst v1.5.3 [29] and ML dating in TreeTime [30] were consistent but had wider intervals compared to the estimates obtained by the least-squares dating in LSD2 [31] and the Gamma-Poisson mixture model in treedater [32], which had the narrowest interval (from 2.3×10^−3^ to 2.4×10^−3^). Therefore, based on our observations, the Gamma-Poisson mixture model in treedater [32] produces the most precise estimates of TMRCA and evolutionary rate of near full-length genomic sequences of HIV-1 subtype C for the four datasets compared to the other three methods.

We were also able to capture the highest number of transmission events when analysing locrisk626, indicating that subsampling by date, country and risk group might produce the best dataset for reconstructing the transmission dynamics of HIV-1 subtype C while curbing potential sampling biases, which is in line with previous findings [25,35, 36].

The analysis presented here also shed light on the epidemiological link of HIV-1 subtype C between the African and European circulating diversity. Travelling between different locations has previously been suggested to shape the geographic distribution of human viruses, such as hepatitis C virus (HCV) [37]. The nature of this link could be a consequence of the tight connectivity between the two continents during the colonial era [38] as well due to the travel history of Europeans to African countries for ‘sex tourism’ as described in a previous study [39]. It has also been hypothesized that the socio-demographic climate of southern Africa between the 1960s and 1990s favoured the rapid expansion of subtype C intra-continentally and across continents to Europe through the SH risk group [13].

Furthermore, our analysis revealed a clustering of Asian, African and American sequences (Fig. 2). We hypothesize that this might have been attributable to two routes of transmissions: firstly, the introduction of subtype C in South America occurred through a founder effect in Brazil, however the exact source remains unclear. This could be partly due to limited sampling or sampling bias. Tulio de Oliveira *et al*. employed Bayesian phylogenetic approaches to a *pol* gene dataset, suggesting that sequences from Brazil, the UK and East Africa were related [40]. Other studies also conclude that the Brazilian subtype C epidemic rose from closely related strains and disseminated to neighbouring countries like Argentina, Uruguay, Paraguay and Venezuela. The timing and the point of entry of subtype C introduction into Brazil as well as the origin of the founder lineage remain controversial due to sampling limitations [41].

Regarding the observed transmission dynamics between Africa and Asia, a recent study from China suggested that the seeding of subtype C from southern Africa into India occurred in 1977, which subsequently exported the virus to China via multiple introductions [42].

In summary, in this study, we use a set of more than a thousand existing full length genome sequences collected in the past four decades in four continents and applied ML phylodynamic tools to investigate the evolution of HIV-1 subtype C and to track the chronology of the risk group and geographical spread via molecular epidemiology. Our study suggests that investigating the evolutionary history and transmission dynamics of the locrisk626 dataset using the Gamma-Poisson mixture model in treedater [32] results in the most likely estimates of the TMRCA and evolutionary rate for near full-length genomic sequences of HIV-1 subtype C. Therefore, we derive that HIV-1 subtype C likely emerged around mid-1939 and has been evolving at a rate of 0.0023 substitutions per site per year. We also estimated that throughout its circulation, at least 71 and 115 introduction events have occurred among continents and risk groups, respectively, mostly from Africa to Europe and among NR and SH risk groups. However, our conclusions are limited by the risk group analysis, due to a large proportion of the sequences being labelled as NR on the reported route of infection. Given the clustering of NR- and SH-labelled viral sequences, we hypothesize that NR-sequences most likely represent the SH risk group, and thus speculate that HIV-1 subtype C is mostly spread through heterosexual transmission, but at the moment there is no way of disentangling how other risk groups might contribute the current viral dynamics.

## supplementary material

10.1099/jmm.0.001827 Uncited Supplementary Material 1.

10.1099/jmm.0.001827 Uncited Table S1.

## Abbreviations

AIC: akaike information criterion
AICc: corrected akaike information criterion
AIDS: acquired immunodeficiency syndrome
ART: anti-retroviral treatment
BIC: Bayesian information criterion
CRFs: circulating recombinant forms
DT: decision theory performance-based selection
GTR: general time-reversible substitution model
HCV: hepatitis C virus
HIV: human immunodeficiency virus
LANL: Los Alamos National Laboratory
MB: mother-baby
ML: maximum-likelihood
NHP: non-human primate
NR: not recorded
OT: the other
PI: IV drug user
SAMPI: sequence sampling tool for phylogenetics
SH: heterosexual
SM: male sex with male
TMRCA: the time to the most recent common ancestor
