# Supplementary material for: The emergence and circulation of human immunodeficiency virus (HIV)-1 subtype C
Source: J Med Microbiol. 2024 May 17;73(5):001827. doi: 10.1099/jmm.0.001827 (PMC11893361; doi:10.1099/jmm.0.001827)
Supplement: Uncited Supplementary Material 1. [file jmm-73-01827-s001.pdf]

# The emergence and circulation of HIV-1 subtype C

Xingguang Li[1], Sana Tamim[2], Nídia S. Trovão[2]

1. Guoke Ningbo Life Science and Health Industry Research Institute, Ningbo, 315000, China.

2. Division of International Epidemiology and Population Studies, Fogarty International Center, National Institutes of Health, Bethesda, Maryland, 20892, USA

Correspondence to:

Xingguang Li, PhD. Guoke Ningbo Life Science and Health Industry Research Institute, Ningbo, 315000, China. E-mail: xingguanglee@hotmail.com.

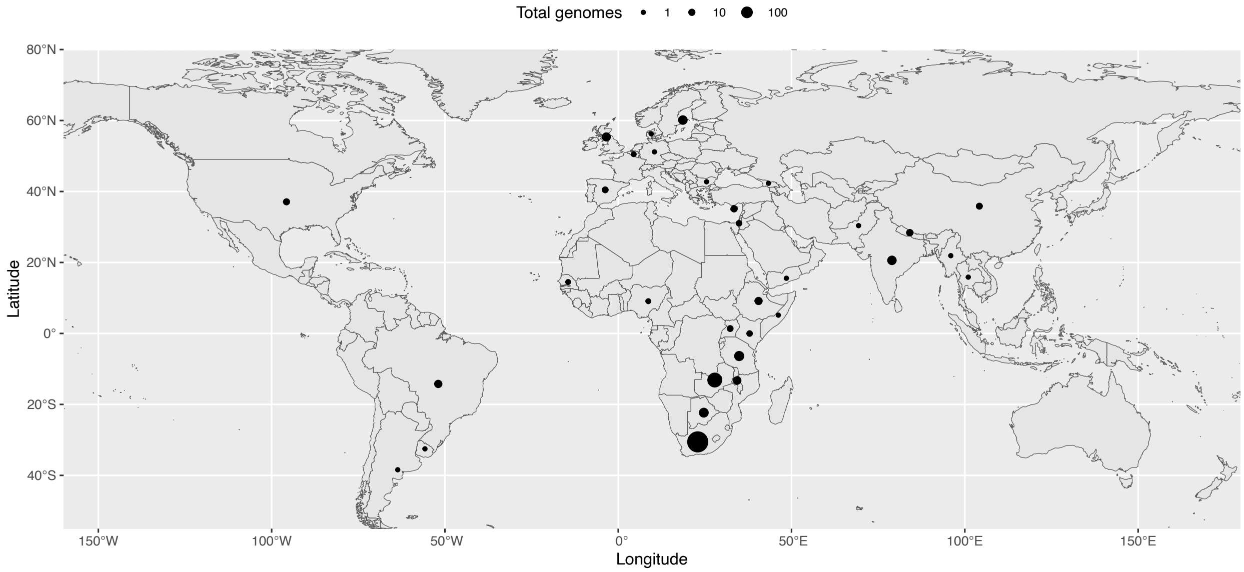


**Supplementary Figure S1. Geographic distribution of SARS-CoV-2 genomes.**

Geographic distribution of HIV-1 subtype C genomes by country. Circle size represents number of HIV-1 subtype C genomes.

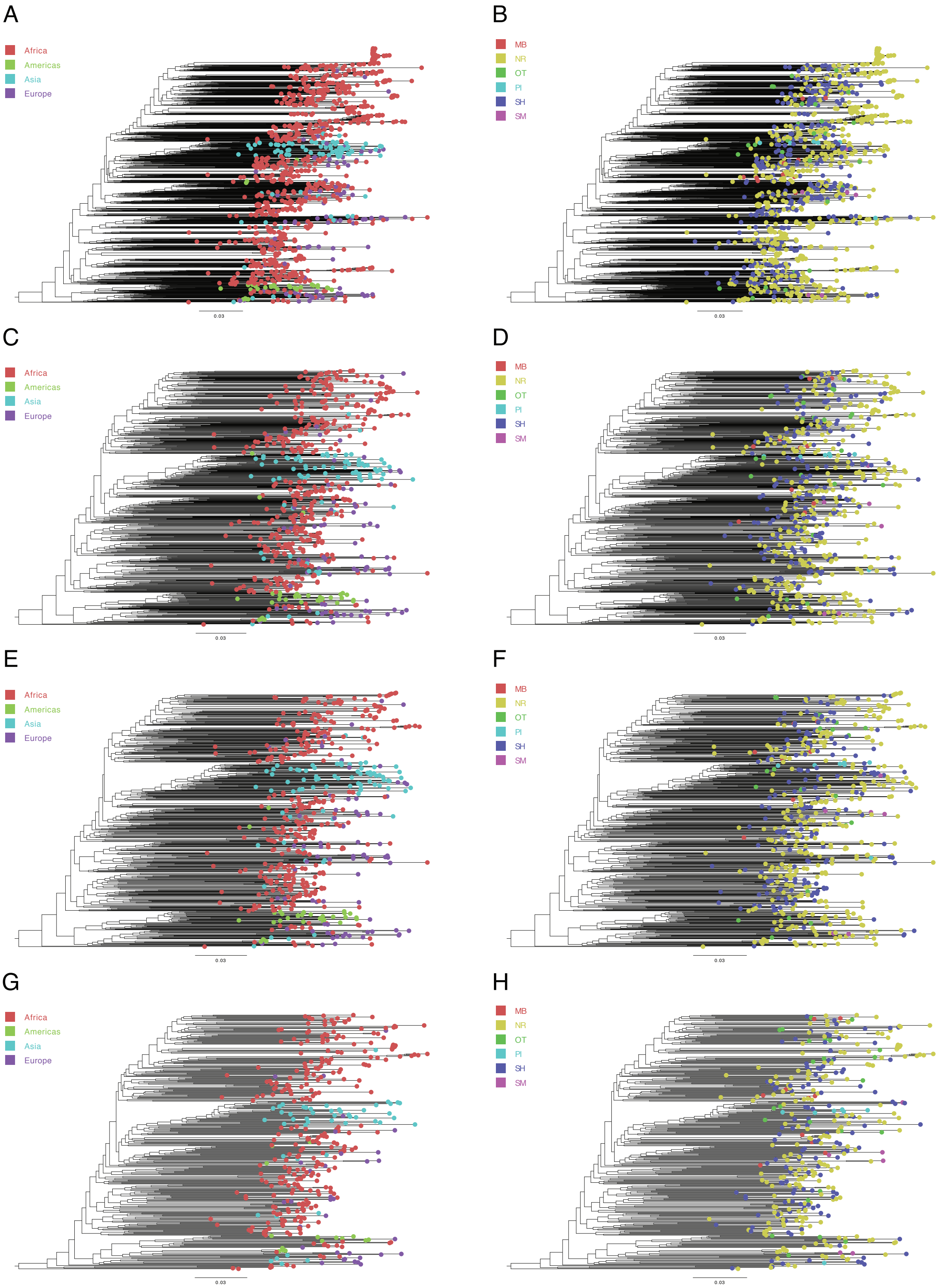
A
Africa
Americas
Asia
Europe
0.03
B
MB
NR
OT
PI
SH
SM
0.03
C
Africa
Americas
Asia
Europe
0.03
D
MB
NR
OT
PI
SH
SM
0.03
E
Africa
Americas
Asia
Europe
0.03
F
MB
NR
OT
PI
SH
SM
0.03
G
Africa
Americas
Asia
Europe
0.03
H
MB
NR
OT
PI
SH
SM
0.03

**Supplementary Figure S2. Estimated ML phylogenetic trees of HIV-1 subtype C.**

Maximum likelihood (ML) phylogenetic trees of HIV-1 subtype C for the four datasets (A, B for full1210; C, D for locrisk626; E, F for loc562; and H, G for risk393). Tip colors indicate sampling locations (A, C, E, and G) and risk groups (B, D, F, and H). Scale bar at the bottom indicates 0.03 nucleotide substitutions per site. SM: male sex with male; PI: IV drug user; SH: heterosexual; MB: mother-baby; NR: not recorded; and OT: the other.

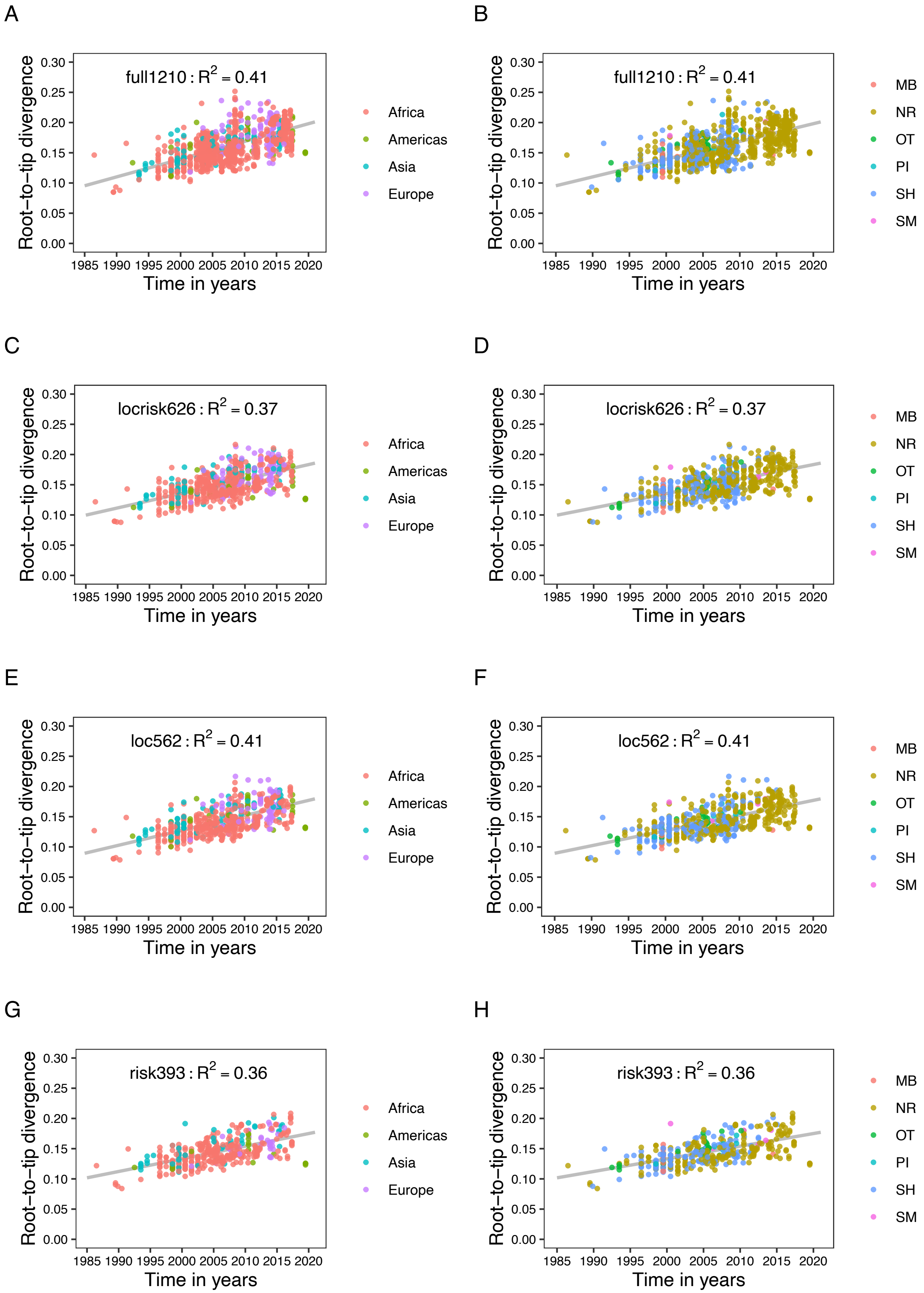
A
full1210 : R2 = 0.41
B
full1210 : R2 = 0.41
C
locrisk626 : R2 = 0.37
D
locrisk626 : R2 = 0.37
E
loc562 : R2 = 0.41
F
loc562 : R2 = 0.41
G
risk393 : R2 = 0.36
H
risk393 : R2 = 0.36
Root−to−tip divergence
Time in years
0.00 0.05 0.10 0.15 0.20 0.25 0.30
1985 1990 1995 2000 2005 2010 2015 2020
Africa
Americas
Asia
Europe
MB
NR
OT
PI
SH
SM

**Supplementary Figure S3. Linear regression plots of root-to-tip genetic divergence against sampling date of HIV-1 subtype C.**

A, B for full1210; C, D for locrisk626; E, F for loc562; and H, G for risk393. Point colors indicate sampling locations (A, C, E, and G) and risk groups (B, D, F, and H). Gray-colored line represents the linear regression line. SM: male sex with male; PI: IV drug user; SH: heterosexual; MB: mother-baby; NR: not recorded; and OT: the other.

A

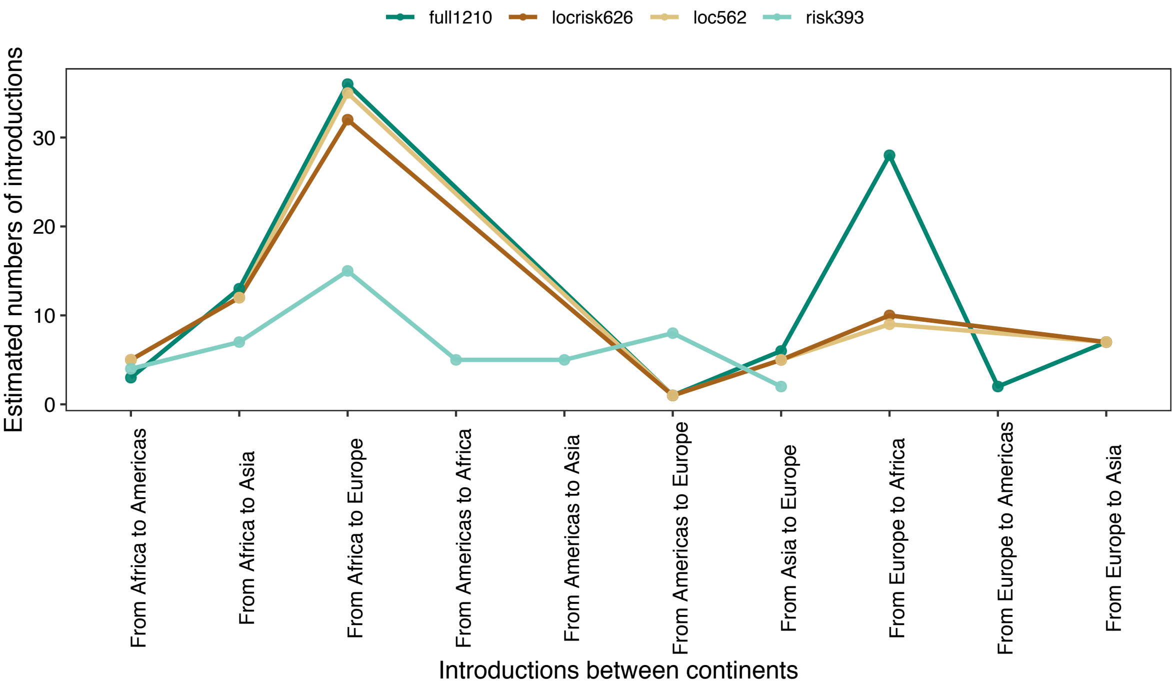

full1210
locrisk626
loc562
risk393
Estimated numbers of introductions
0
10
20
30
From Africa to Americas
From Africa to Asia
From Africa to Europe
From Americas to Africa
From Americas to Asia
From Americas to Europe
From Asia to Europe
From Europe to Africa
From Europe to Americas
From Europe to Asia
Introductions between continents


B

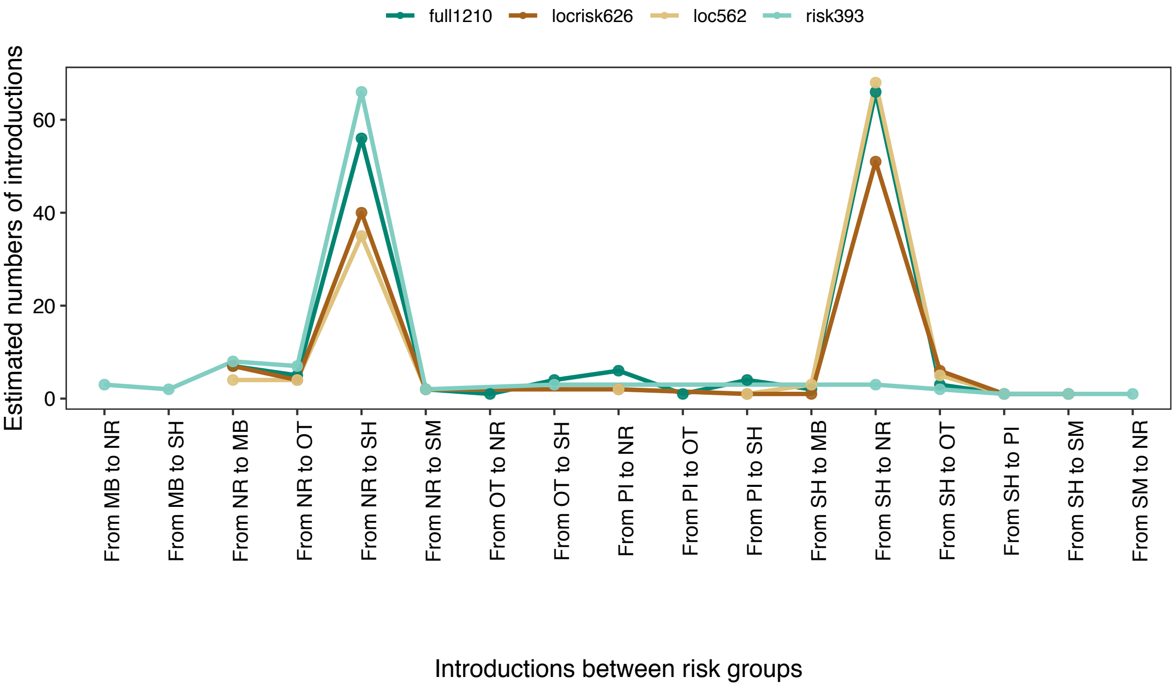

full1210
locrisk626
loc562
risk393
Estimated numbers of introductions
0
20
40
60
From MB to NR
From MB to SH
From NR to MB
From NR to OT
From NR to SH
From NR to SM
From OT to NR
From OT to SH
From PI to NR
From PI to OT
From PI to SH
From SH to MB
From SH to NR
From SH to OT
From SH to PI
From SH to SM
From SM to NR
Introductions between risk groups

**Supplementary Figure S4. Estimated number of introductions of HIV-1 subtype C.**

(A) Estimated number of introductions among continents for the four datasets (full1210, locrisk626, loc562, and risk393). (B) Estimated number of introductions among risk groups for the four datasets (full1210, locrisk626, loc562, and risk393). SM: male sex with male; PI: IV drug user; SH: heterosexual; MB: mother-baby; NR: not recorded; and OT: the other.

**Supplementary Table S1 - List of sequence information analysed in the present study.**

| Accession number |
|---|
| AB023804 |
| AB097871 |
| AB254141 |
| AB254142 |
| AB254143 |
| AB254146 |
| AB254148 |
| AB254149 |
| AB254150 |
| AB254155 |
| AB254156 |
| AB485645 |
| AF067154 |
| AF067155 |
| AF067157 |
| AF067158 |
| AF067159 |
| AF110959 |
| AF110962 |
| AF110967 |
| AF110969 |
| AF110972 |
| AF110973 |
| AF110976 |
| AF110979 |
| AF286223 |
| AF286224 |
| AF286225 |
| AF286227 |
| AF286228 |
| AF286231 |
| AF286232 |
| AF286233 |
| AF286234 |
| AF286235 |
| AF290027 |
| AF361874 |
| AF361875 |
| AF411966 |
| AF411967 |

AF443074
AF443075
AF443076
AF443077
AF443078
AF443079
AF443080
AF443081
AF443082
AF443083
AF443084
AF443085
AF443086
AF443087
AF443088
AF443089
AF443090
AF443091
AF443092
AF443093
AF443094
AF443095
AF443096
AF443097
AF443098
AF443099
AF443100
AF443101
AF443102
AF443103
AF443104
AF443105
AF443107
AF443108
AF443109
AF443110
AF443111
AF443112
AF443113
AF443114
AF443115
AF457054
AY043173

AY043174
AY043175
AY043176
AY049708
AY118165
AY118166
AY158533
AY158534
AY158535
AY162223
AY162224
AY162225
AY228556
AY228557
AY253303
AY253304
AY253307
AY253308
AY253310
AY253312
AY253313
AY253317
AY253320
AY253321
AY253322
AY255823
AY255824
AY255825
AY255826
AY444800
AY444801
AY463217
AY463218
AY463219
AY463220
AY463221
AY463222
AY463223
AY463224
AY463225
AY463226
AY463227
AY463228

AY463229
AY463230
AY463231
AY463232
AY463233
AY463234
AY463236
AY463237
AY563169
AY563170
AY585264
AY585265
AY585266
AY585267
AY585268
AY703908
AY703909
AY703910
AY703911
AY713413
AY713414
AY713415
AY713416
AY713417
AY727522
AY727523
AY727524
AY727525
AY734550
AY734551
AY734556
AY734558
AY734559
AY734560
AY772690
AY772691
AY772692
AY772693
AY772694
AY772695
AY772696
AY772698
AY772699

AY772700
AY795906
AY878054
AY878055
AY878056
AY878057
AY878058
AY878059
AY878060
AY878061
AY878062
AY878063
AY878064
AY878065
AY878068
AY878070
AY878071
AY878072
AY901965
AY901966
AY901967
AY901968
AY901969
AY901970
AY901971
AY901972
AY901973
AY901974
AY901975
AY901976
AY901977
AY901978
AY901979
AY901980
AY901981
AY945738
AY967806
DQ011165
DQ011166
DQ011167
DQ011169
DQ011170
DQ011171

DQ011172
DQ011173
DQ011174
DQ011175
DQ011176
DQ011177
DQ011178
DQ011179
DQ011180
DQ056404
DQ056405
DQ056406
DQ056408
DQ056409
DQ056410
DQ056411
DQ056412
DQ056413
DQ056414
DQ056415
DQ056416
DQ056417
DQ056418
DQ093585
DQ093586
DQ093587
DQ093588
DQ093589
DQ093590
DQ093591
DQ093592
DQ093593
DQ093594
DQ093595
DQ093596
DQ093597
DQ093598
DQ093599
DQ093600
DQ093601
DQ093602
DQ093604
DQ093605

DQ093607
DQ164104
DQ164106
DQ164107
DQ164108
DQ164109
DQ164110
DQ164111
DQ164113
DQ164114
DQ164115
DQ164117
DQ164118
DQ164119
DQ164121
DQ164122
DQ164126
DQ164127
DQ164129
DQ207941
DQ275642
DQ275643
DQ275644
DQ275645
DQ275646
DQ275647
DQ275648
DQ275649
DQ275650
DQ275651
DQ275652
DQ275653
DQ275654
DQ275655
DQ275656
DQ275657
DQ275658
DQ275659
DQ275660
DQ275661
DQ275664
DQ351216
DQ351217

DQ351218
DQ351219
DQ351220
DQ351221
DQ351222
DQ351223
DQ351224
DQ351225
DQ351226
DQ351227
DQ351228
DQ351229
DQ351230
DQ351231
DQ351232
DQ351234
DQ351235
DQ351237
DQ369976
DQ369977
DQ369978
DQ369979
DQ369980
DQ369981
DQ369982
DQ369983
DQ369984
DQ369985
DQ369986
DQ369987
DQ369988
DQ369989
DQ369990
DQ369991
DQ369992
DQ369993
DQ369994
DQ369995
DQ369996
DQ369997
DQ396364
DQ396365
DQ396367

DQ396368
DQ396369
DQ396370
DQ396371
DQ396372
DQ396373
DQ396374
DQ396375
DQ396376
DQ396377
DQ396378
DQ396380
DQ396381
DQ396382
DQ396383
DQ396384
DQ396385
DQ396386
DQ396387
DQ396388
DQ396389
DQ396390
DQ396391
DQ396392
DQ396393
DQ396394
DQ396395
DQ396399
DQ445632
DQ445633
DQ445634
DQ445635
DQ445637
EF469243
EF514713
EU293444
EU293445
EU293446
EU293447
EU293448
EU293449
EU293450
EU786673

EU786681
EU884500
FJ388901
FJ388913
FJ388948
FJ388952
FJ496185
FJ496195
FJ670521
GQ999972
GQ999973
GQ999974
GQ999975
GQ999976
GQ999977
GQ999978
GQ999979
GQ999980
GQ999981
GQ999982
GQ999983
GQ999984
GQ999985
GQ999986
GQ999987
GQ999988
GQ999989
GQ999990
GQ999991
JF683740
JF683755
JF683757
JF683768
JF683770
JF683776
JF683777
JN188292
JN692434
JX140663
JX140664
JX140666
JX140667
JX140668

JX140669
JX974244
JX974245
JX974246
KC156114
KC156116
KC156117
KC156120
KC156122
KC156125
KC156127
KC156130
KC156210
KC156211
KC156212
KC156213
KC156215
KC156216
KC156218
KC156219
KC156220
KC156221
KC898980
KC898995
KC898996
KF250403
KF250404
KF526207
KF527172
KF716466
KF716467
KF766537
KF766540
KF766541
KF835515
KF835522
KP109480
KP109481
KP109482
KP109483
KP109484
KP109485
KP109486

KP109487
KP109488
KP109489
KP109494
KP109495
KP109496
KP109516
KP109517
KP109520
KP109521
KP109522
KP109523
KP109524
KP109525
KP109526
KP109527
KP411830
KP411831
KP411832
KP411833
KP411834
KP411835
KP411836
KP411837
KP411839
KR820294
KR820314
KR820324
KR820326
KR820341
KR820358
KR820367
KR820385
KR820394
KR820415
KR820422
KR820440
KT022362
KT022366
KT022371
KT124786
KT183051
KT183056

KT183063
KT183068
KT183078
KT183084
KT183089
KT183094
KT183103
KT183123
KT183128
KT183135
KT183140
KT183145
KT183150
KT183155
KT183168
KT183172
KT183178
KT183188
KT183196
KT183201
KT183208
KT183213
KT183218
KT183227
KT183239
KT183245
KT183250
KT183252
KT183258
KT183264
KT183271
KT183274
KT183279
KT183289
KT183301
KT183336
KT276258
KT427674
KT427677
KT427678
KT427736
KT427752
KT427800

KT427806
KT427810
KT427817
KT982201
KU168308
KU319529
KU319530
KU319531
KU319533
KU319534
KU319535
KU319536
KU319537
KU319538
KU319540
KU319541
KU319542
KU319543
KU319544
KU319545
KU319546
KU319548
KU319549
KU319551
KU341722
KU341723
KU341724
KU341725
KU341726
KU341728
KU341730
KU341731
KU749412
KU749415
KU749416
KU749417
KU749425
KU749426
KX069219
KX069220
KX069221
KX069222
KX069223

KX069224
KX069225
KX069226
KX069227
KX069228
KX228799
KX228818
KX228821
KX389612
KX907337
KX907338
KX907339
KX907345
KX907349
KX907356
KX907358
KX907363
KX907366
KX907368
KX907374
KX907375
KX907380
KX907382
KX907384
KX907385
KX907388
KX907390
KX907391
KX907392
KX907393
KX907394
KX907395
KX907398
KX907399
KX907400
KX907405
KX907407
KX907413
KX907415
KX907416
KX907420
KX907422
KX907424

KX907425
KX907426
KX907433
KY111987
KY112082
KY112197
KY112251
KY112261
KY112360
KY112577
KY496624
KY658705
KY658706
KY713228
KY713229
KY713230
KY713231
KY713233
KY713234
KY713235
KY713236
KY989952
MF109366
MF109367
MF109379
MF109387
MF109389
MF109403
MF109413
MF109435
MF109437
MF109441
MF109504
MF109507
MF109510
MF109513
MF109522
MF109528
MF109535
MF109539
MF109557
MF109563
MF109589

MF109591
MF109592
MF109593
MF109599
MF109606
MF109607
MF109609
MF109612
MF109617
MF109626
MF109670
MF109676
MF109684
MF109695
MF109706
MF109707
MF373126
MF373132
MF373133
MF373134
MF373135
MF373136
MF373137
MF373139
MF373145
MF373146
MF373148
MF373152
MF373154
MF373156
MF373157
MF373158
MF373159
MF373164
MF373165
MF373166
MF373169
MF373170
MF373171
MF373175
MF373176
MF373177
MF373178

MF373179
MF373184
MF373187
MF373192
MF373193
MF373197
MF373198
MF373199
MF373202
MG989567
MH705137
MH933706
MH933707
MH933714
U46016
U52953
KU319547
MH746257
MH933704
MH933705
MH933708
MH933709
MH933710
MH933711
MH933713
MK041550
MK041561
MK041563
MK041564
MK041576
MK493076
MK493077
MK493078
MK493079
MK643537
MK643538
MK643539
MK643540
MK643541
MK643542
MK643543
MK643544
MK643545

MK643546
MK643547
MK643548
MK643549
MK643550
MK643551
MK643559
MK643564
MK643565
MK643569
MK643571
MK643572
MK643573
MK643575
MK643577
MK643581
MK643584
MK643585
MK643586
MK643588
MK643591
MK643598
MK643600
MK643602
MK643604
MK643608
MK643610
MK643611
MK643613
MK643614
MK643615
MK643616
MK643618
MK643620
MK643622
MK643624
MK643629
MK643631
MK643632
MK643639
MK643641
MK643650
MK643653

MK643654
MK643655
MK643656
MK643660
MK643661
MK643663
MK643664
MK643669
MK643671
MK643672
MK643674
MK643676
MK643677
MK643679
MK643686
MK643687
MK643690
MK643692
MK643697
MK643698
MK643705
MK643707
MK643709
MK643713
MK643715
MK643718
MK643719
MK643729
MK643730
MK643732
MK643734
MK643736
MK643740
MK643743
MK643744
MK643746
MK643754
MK643756
MK643759
MK643760
MK643761
MK643764
MK643766

MK643771
MK643776
MK643779
MK643780
MK643782
MK643783
MK643785
MK643787
MK643788
MK643791
MK643792
MK643794
MK643797
MK643802
MK643803
MN097551
MN097552
MN097553
MN097554
MN097555
MN097556
MN097557
MN097558
MN097559
MN097560
MN097561
MN097562
MN097563
MN097564
MN097565
MN097566
MN097567
MN097568
MN097569
MN097570
MN097571
MN097572
MN097573
MN097574
MN097575
MN097576
MN097577
MN097578

MN097579
MN097580
MN097581
MN097582
MN097583
MN097584
MN097585
MN097586
MN097587
MN097588
MN097589
MN097590
MN097591
MN097592
MN097593
MN097594
MN097595
MN097596
MN097597
MN097598
MN097599
MN097600
MN097601
MN097602
MN097603
MN097604
MN097605
MN097608
MN097609
MN097610
MN097611
MN097612
MN097617
MN097618
MN097619
MN097620
MN097621
MN097622
MN097623
MN097630
MN097631
MN097632
MN097633

MN097634
MN097635
MN097636
MN097637
MN097638
MN097639
MN097645
MN097646
MN097647
MN097648
MN097649
MN097650
MN097651
MN097652
MN097653
MN097654
MN097655
MN097656
MN097657
MN097658
MN097659
MN097660
MN097661
MN097662
MN097663
MN097664
MN097665
MN097666
MN097667
MN097668
MN097669
MN097670
MN097671
MN097672
MN097673
MN097674
MN097675
MN097676
MN097677
MN097678
MN097679
MN097680
MN097681

MN097682
MN097683
MN097684
MN097685
MN097686
MN097687
MN097688
MN097689
MN097690
MN097691
MN097692
MN097693
MN097694
MN097695
MN097696
MN202471
MN202472
MN485984
MN486011
MN611462
MN611463
MN611464
MN611465
MN611466
MN611467
MN611468
MN650390
MN650417
MN650468
MN650469
MN650550
MN650551
MN650552
MN703145
MN703146
MN703147
MN703148
MN703149
MN703150
MT194138
MT194167
MT194168
MT194169

MT194170
MT194171
MT194172
MT194173
MT194174
MT194175
MT194176
MT194177
MT194178
MT194282
MT194283
MT194284
MT194285
MT194286
MT194287
MT194288
MT194289
MT194305
MT194314
MT194315
MT194316
MT194317
MT194318
MT194319
MT194320
MT194321
MT194322
MT194323
MT194324
MT194325
MT194326
MT194327
MT194338
MT194343
MT194344
MT194468
MT194478
MT194481
MT194482
MT194483
MT194484
MT194485
MT194488

MT194489
MT194490
MT194491
MT194492
MT194493
MT194494
MT194495
MT194496
MT194497
MT194498
MT194600
MT194601
MT194602
MT194603
MT194604
MT194605
MT194606
MT194607
MT194608
MT194609
MT194610
MT194738
MT194739
MT194740
MT194741
MT194742
MT194743
MT194744
MT194745
MT194746
MT194747
MT194748
MT194749
MT194782
MT194792
MT194854
MT194855
MT194856
MT194857
MT194858
MT194859
MT194860
MT194861

MT194862
MT194863
MT194864
MT194865
MT194866
MT194867
MT194868
MT194869
MT194870
MT194871
MT194974
MT194983
MT194984
MT194985
MT194986
MT194987
MT194988
MT194989
MT194990
MT194991
MT194992
MT195134
MT195135
MT195136
MT195137
MT195138
MT195139
MT195140
MT195141
MT195142
MT195143
MT195144
MT195145
MT195146
MT195147
MT195148
MT195149
MT195150
MT195151
MT195152
MT195153
MT195154
MT195155

MT195156
MT195232
MT195233
MT195234
MT195235
MT195236
MT195237
MT195238
MT195239
MT195240
MT195241
MT195378
MT195387
MT195388
MT195389
MT195390
MT195391
MT195392
MT195393
MT195394
MT195395
MT195396
MT195397
MT195398
MT195399
MT195400
MT195401
MT195402
MT195403
MT195493
MT195494
MT195495
MT195496
MT195497
MT195498
MT195499
MT195500
MT195501
MT195502
MT195503
MT195504
MT195505
MT195506

| |
|---|
| MT195507 |
| MT195508 |
| MT195509 |
| MT195510 |
| MT195511 |
| MT195512 |
| MT195513 |
| MT195514 |
| MT195515 |
| MT195527 |
| MT222958 |
| MT347678 |
| MT347679 |
| MT347680 |
| MT347681 |
| MW262771 |
| MW262772 |
| MW262773 |
| MW262774 |
| MW262775 |

**Supplementary Table S2 - Degree of sample overlap ('sharedness') between datasets.**

| | full1210 | locrisk626 | loc562 | risk393 |
|---|---|---|---|---|
| **full1210** | - | - | - | - |
| **locrisk626** | 626 (51.7%) | - | - | - |
| **loc562** | 562 (46.4%) | 503 (80.4) | - | - |
| **risk393** | 393 (32.5%) | 359 (57.3%) | 329 (58.5%) | - |
